# Intersubband terahertz transitions in Landau level system of cascade GaAs/AlGaAs quantum well structures in strong tilted magnetic field

**DOI:** 10.1186/1556-276X-7-491

**Published:** 2012-08-31

**Authors:** Maksim P Telenkov, Yury A Mityagin, Petr F Kartsev

**Affiliations:** 1 Solid State Physics Dept., P.N.Lebedev Physical Institute, Moscow 119991, Russia; 2 Theoretical Physics and Quantum Technologies Dept., Moscow Institute for Steel and Alloys, Moscow 119049, Russia; 3 National Research Nuclear Center MEPHI, Moscow 115409, Russia

**Keywords:** Quantum well structures, Landau levels, terahertz transitions.

## Abstract

The tunable terahertz intersubband Landau level transitions in resonant tunneling cascade quantum well structures are considered. The way of lifting the selection rule forbidding the inter-Landau level terahertz transitions of interest by applying a magnetic field tilted with respect to the structure layers is proposed. The importance of asymmetric structure design to achieve considerable values of transition dipole matrix elements is demonstrated.

## Background

**Figure 1:**
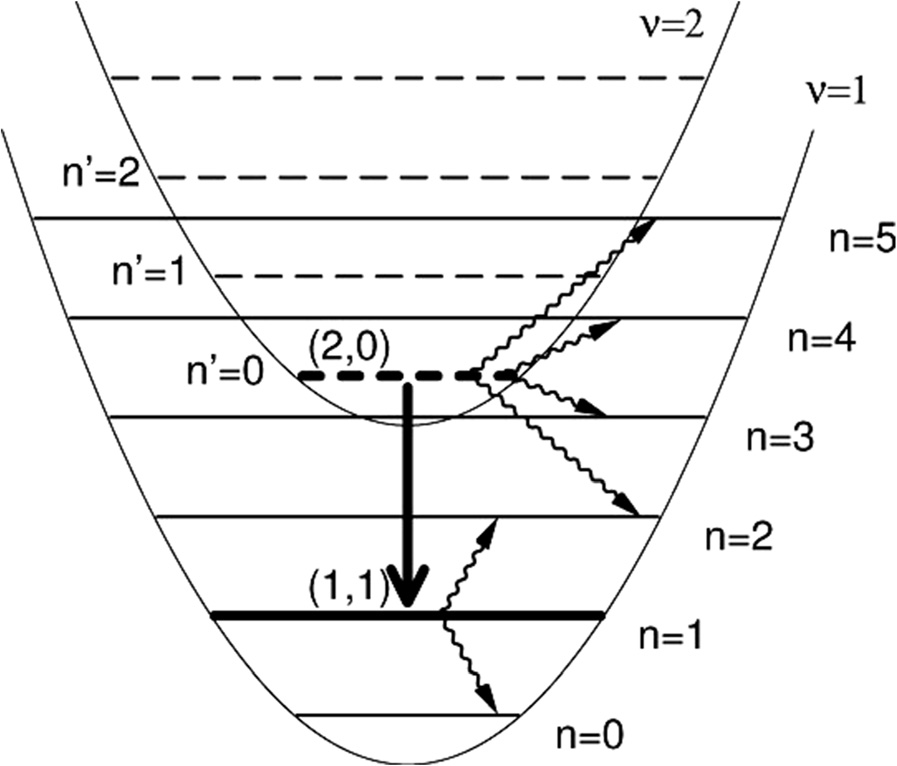
**A scheme of transitions between Landau levels in a quantum well.** The thick arrow indicates the (2, 0) → (1,1) radiative transition, and the wavy arrows mark the transitions due to the electron–electron scattering.

Recently, the possibility to achieve a population inversion in the system of Landau levels (LL) in cascade quantum well structures in strong magnetic field under a condition of sequential resonant tunneling, i.e., in strong transverse electric field, was shown [1]. If the spacing between the first and any upper (*ν*th) subbands is lower than the optical phonon energy (i.e., when the optical phonon scattering is suppressed), the population of zeroth LL in *ν*th subband can exceed that of the first LL in the first subband. So, the stimulated emission of terahertz radiation can be achieved on the transitions between these LLs, and the emission frequency may continuously be tuned in a wide range of terahertz frequencies by the variation of the magnetic field strength according to the relation

(1) $$\hbar \omega =\Delta E_{1\nu }-\hbar \omega_{\text{c}}\text{,}$$

where $\Delta E_{1\nu }$ is the subband spacing and *ω*_*c*_ is the cyclotron frequency. A scheme of transitions between Landau levels of subbands 1 and 2 in a quantum well structure considered in [1] is shown in Figure 1. The main problem arising is that in a magnetic field directed perpendicularly to the structure layers, the optical transition of interest, shown in Figure 1, is forbidden, i.e., the corresponding dipole matrix element is exactly equal to zero.

In [1], we proposed a possible way to overcome the difficulty and to provide nonzero matrix element values for transitions of interest by tilting the magnetic field with respect to the structure layers. In the present paper, we investigated the effect of magnetic field tilt on the optical matrix element of the intersubband Landau level transitions. The importance of an asymmetric structure design to achieve substantial values of transition dipole matrix elements was revealed, and an asymmetric two-well periodic structure was proposed as a possible solution maximizing the optical matrix element of the terahertz transitions of interest.

## Methods

### Theoretical background

Let us consider the electron in the quantum well structure in the tilted magnetic field $B=B_{∥}e_{y}+B_{⊥}e_{z}$, where z is the growth axis. In Landau gauge $A=\left(B_{∥}z-B_{⊥}y\right)e_{x}$, the electron envelope wave function is given by [2]

(2) $$\psi \left(x,y,z\right)=\frac{\exp\left(ikx\right)}{\sqrt{L}}\cdot f\left(y-\ell_{⊥}^{2}k,z\right)$$

where component $f\left(y,z\right)$ is determined by a two-dimensional Schroedinger equation

(3) $$H_{2\text{D}}f\left(y,z\right)=Ef\left(y,z\right)$$

with Hamiltonian

(4) $$H_{2\text{D}}=H_{⊥}+H_{\text{tilt}}$$

where

(5) $$H_{⊥}=-\frac{\partial }{\partial z}\frac{\hbar^{2}}{2m\left(z\right)}\frac{\partial }{\partial z}+V_{\text{QW}}\left(z\right)-\frac{\hbar^{2}}{2m\left(z\right)}\frac{\partial^{2}}{\partial y^{2}}+\frac{\hbar^{2}}{2m\left(z\right)}\frac{y^{2}}{\ell_{⊥}^{4}}$$

is the Hamiltonian for the case of magnetic field $B={В}_{⊥}e_{z}$ normal to the structure layers, and

(6) $$H_{\text{tilt}}=\frac{\hbar^{2}}{2m\left(z\right)}\cdot \frac{z^{2}}{\ell_{∥}^{4}}-\frac{\hbar^{2}}{m\left(z\right)\ell_{⊥}^{2}\ell_{∥}^{2}}\cdot yz\text{.}$$

Here, $V_{\text{QW}}\left(z\right)$ is the quantum well potential, $m\left(z\right)=\{\begin{array}{l} m_{w},z\in \text{w}ell\;\\ m_{b},z\in \text{b}arrier \end{array}$ is the effective mass, $\ell_{⊥}=\sqrt{\frac{\hbar c}{eB_{⊥}}}$ and $\ell_{∥}=\sqrt{\frac{\hbar c}{eB_{∥}}}$ are the magnetic lengths for transverse (B_⊥_) and longitudinal (B_∥_) magnetic field components and L is the thickness of the structure.

In the case of the magnetic field $B={}_{⊥}e_{z}$ being normal to the structure layers, the variables in the Schroedinger equation are separated, and energy levels and electron wave functions are given by the expressions [3]

(7) $$E{}_{\left(\nu ,n\right)}=\epsilon_{\nu }+\hbar \omega_{⊥}\cdot \left(n+1/2\right)$$

and

(8) $$f{}_{\left(\nu ,n\right)}\left(y,z\right)=\phi_{\nu }\left(z\right)\Phi_{n}\left(y\right)\text{,}$$

where $\Phi_{n}\left(y\right)$ is the wave function of harmonic oscillator with mass $m_{w}$ and frequency $\omega_{⊥}=eB_{⊥}/\left(m_{w}c\right)$, and $\epsilon_{\nu }$ and $\phi_{\nu }\left(z\right)$ are the energy and wave function of νth subband. Here, the small effect of the effective lowering of the barrier height with the increasing of the Landau level number n [3] is neglected.

It can be easily seen that in this case, the dipole matrix element

(9) $$D_{\left(2,0)\to (1,1\right)}=\langle \frac{\exp\left(ik_{1}x\right)}{\sqrt{L}}f_{\left(2,0\right)}\left(y-\ell_{⊥}^{2}k_{1},z\right)|r|\frac{\text{exp}\left(ik_{1}x\right)}{\sqrt{L}}f_{\left(1,1\right)}\left(y-\ell_{⊥}^{2}k_{1},z\right)\rangle$$

is exactly equal to zero for any polarization due to the orthogonality of subband ($\langle \phi_{\nu_{1}}|\phi_{\nu_{2}}\rangle =\delta_{\nu_{1},\nu_{2}}$) and oscillator ($\langle \Phi_{n_{1}}|\Phi_{n_{2}}\rangle =\delta_{n_{1},n_{2}}$) wave functions, that is, the considered (2,0) → (1,1) transition is optically forbidden.

However, the matrix element of the specified transition can be made nonzero by applying an additional component $B_{║}$ of the magnetic field parallel to the layers, that is, by tilting the magnetic field with respect to the structure layers. Now, due to an additional term

(10) $$H_{\text{mix}}=-\frac{\hbar^{2}}{m\left(z\right)}\frac{yz}{\ell_{∥}^{2}\ell_{⊥}^{2}}$$

arising in Equation 4, the variables in the Schroedinger equation are no longer separated, resulting in the mixing of in-plane and out-of-plane electron motions [4] and lifting of the above selection rule. The effect is similar to the violation of the Δ*n* = 0 selection rule for the resonant tunneling transitions between the Landau levels in the tilted magnetic field [4-10].

Here, we will consider the situation when the matrix element of the Hamiltonian (Equation 6) over the first and second subband stated (Equation 8) is much lower than the subband spacing. This is the case in the magnetic field range when $\hbar \omega_{⊥}<\Delta E_{12}$. The structure of a single-electron spectrum in the tilted magnetic field in this case does not change significantly [4-10]. The main effect of $B_{∥}$ is the shift of the harmonic oscillator center in Equation 8 by the value $\frac{\ell_{⊥}^{2}}{\ell_{∥}^{2}}{\langle z\rangle }_{\nu }$[7], where ${\langle z\rangle }_{\nu }=\int dz{\left|\phi_{\nu }\left(z\right)\right|}^{2}z$ is the average value of the electron coordinate along the z axis in the νth subband state:

(11) $$f_{\left(\nu ,n\right)}\left(y,z\right)=\phi_{\nu }\left(z\right)\Phi_{n}\left(y-\frac{\ell_{⊥}^{2}}{\ell_{∥}^{2}}{\langle z\rangle }_{\nu }\right)\text{.}$$

Substituting wave functions (Equation 11) into Equation 9, the following expression can be obtained for the squared modulus of the dipole matrix element:

(12) $${\left|D_{\left(2,0\right)\to \left(1,1\right)}\right|}^{2}=\delta_{k_{1},k_{2}}\cdot {\left|\langle \phi_{2}\left(z\right)|z|\phi_{1}\left(z\right)\rangle \right|}^{2}\cdot G\left(\xi \right)\text{,}$$

where

(13) $$G\left(\xi \right)=\xi^{2}/2\cdot \exp\left(-\xi^{2}/2\right)\text{,}$$

(14) $$\xi =\left[{\langle z\rangle }_{2}-{\langle z\rangle }_{1}\right]\cdot \frac{\ell_{⊥}}{\ell_{∥}^{2}}\text{.}$$

From this expression, one can see that the dipole matrix element becomes nonzero only if the values ${\langle z\rangle }_{1}$ and ${\langle z\rangle }_{2}$ are substantially different.

In symmetric well potential $V_{\text{QW}}\left(z\right)$, the subband wave functions $\phi_{\nu }\left(z\right)$ are symmetric or antisymmetric with respect to symmetry center of the potential, and the averages (z) are the same for all subbands. So, in symmetric potential, the transition matrix element continues to be close to zero even in the tilted magnetic field. Thus, to provide a nonzero dipole matrix element for transitions of interest along with the application of the tilted magnetic field, it is necessary to introduce an asymmetric potential along the direction of the structure growth.

## Results and discussion

**Figure 2:**
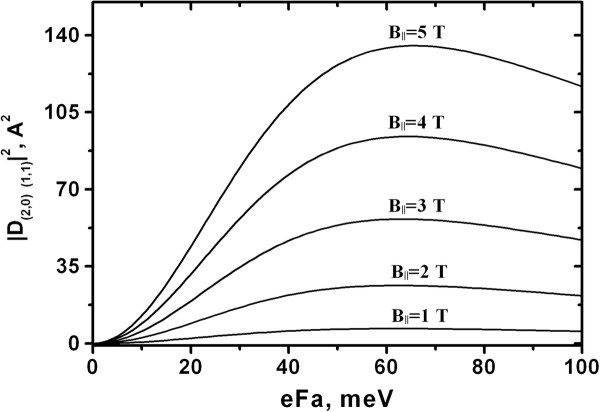
**Calculated dependencies of the dipole matrix element on the applied electric field.** Squared modulus of the dipole matrix element | **D**_(2,0)→(1,1)_|^2^ versus the voltage drop *eFa * per quantum well for different values of the parallel component of the magnetic field *B *_*∥*_ = 1 to 5 T. The calculation was performed for the 250-Å GaAs/Al_0.3_ Ga_0.7_As quantum well. The transverse component of the magnetic field is *B *_*⊥*_ = 5 T.

The simplest solution is to apply an electric field along the structure growth axis especially as the electric field is necessary to provide the resonant tunneling pumping of the LLs of the upper subband. In Figure 2, the calculated dependence of the dipole matrix element of the (2,0) → (1,1) transition in the GaAs/AlGaAs quantum well on the applied electric field is shown. It can be seen that the application of the electric field results in the nonzero dipole matrix element. Nevertheless, since the possible values of the electric field strength are determined by resonant tunneling conditions and cannot be selected independently, this way of providing a nonzero matrix element is not very effective.

**Figure 3:**
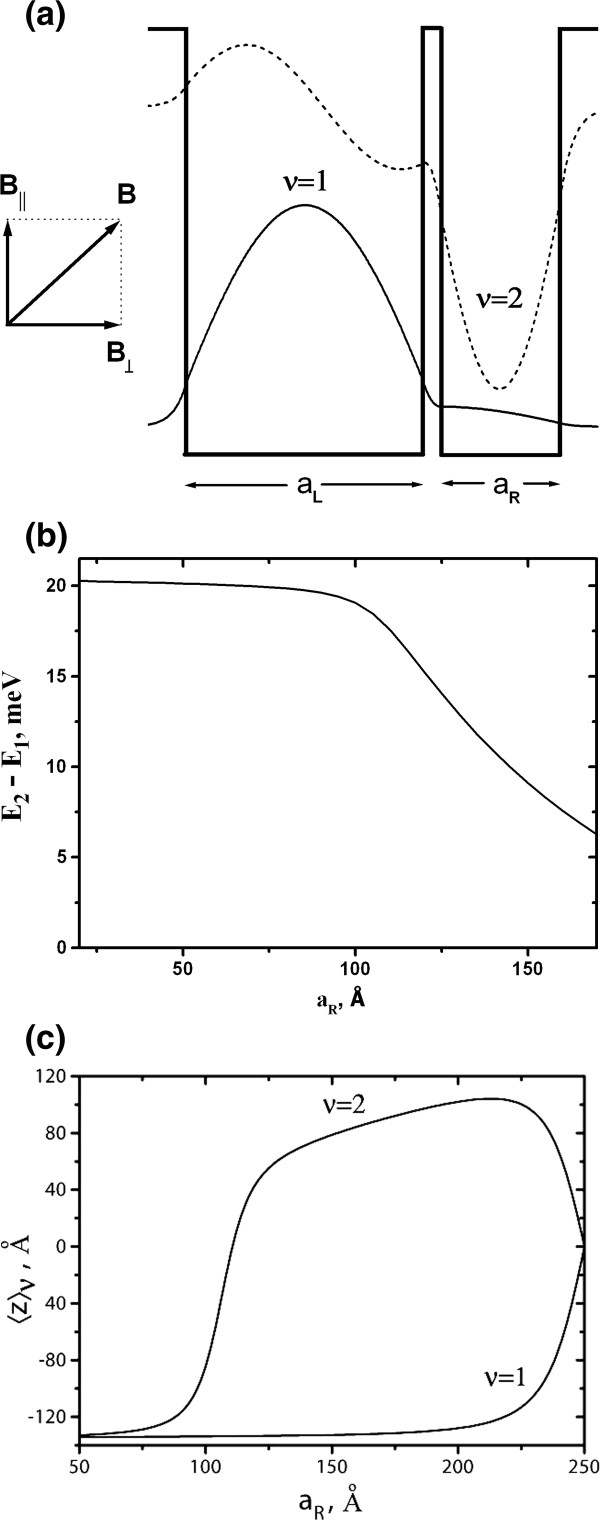
**An asymmetric double well as an active element in periodic cascade quantum well structure. **( **a**) Proposed design of the active element of the periodic cascade quantum well structure and the calculated wave functions of the first and second subbands. ( **b**) The dependence of the 1 to 2 intersubband spacing on the narrow well width a_R_. (**c**) The values of the averages 〈z〉_ν_ for corresponding subbands as a function of narrow well width a_R_.

More effective is the use of an asymmetric design of the structures themselves. One of the possible solutions is to introduce an asymmetric double well as an active element of the periodic cascade quantum well, consisting of two strongly coupled wells with different widths (Figure 3a), as an active element of the periodic cascade quantum well structure. In the asymmetric double quantum well, the first subband wave function is located mainly in the wider well, while the second subband wave function is shifted to the narrower well (Figure 3b). As a result, a significant difference between average coordinates ${\langle z\rangle }_{1}$ and ${\langle z\rangle }_{2}$ is achieved (Figure 3c).

**Figure 4:**
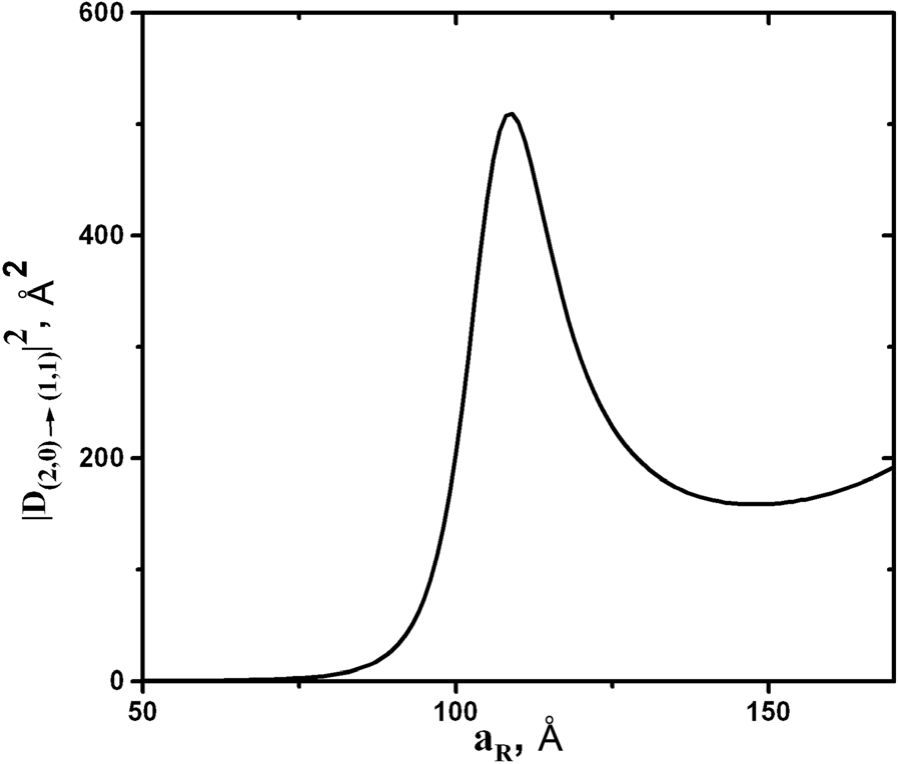
**The calculated dependence of the squared modulus of the dipole matrix element |D**_**(2,0)→(1,1)**_**|**^**2**^**.** Calculated for the double-well structure shown in Figure 3a as a function of narrow well width a_R_.

The dipole matrix element for transitions between the zeroth LL of the second subband and the first LL of the first subband is presented in Figure 4 as a function of narrow quantum well width a_R._. Here, the width of wider well is fixed, while the width a_R_ of the narrow well is varied. A pronounced maximum can be seen at a_R_ = 110 Å, and the maximum achievable value of |**D**_(2,0)→(1,1)_|^2^ is considerably higher than that in previously considered case of the single symmetric quantum well in transverse electric field (see Figure 2 for comparison).

Of course, the structure considered is an example proposed here to illustrate the general way of how the selection rule forbidding the transitions of interest can be overcome. More detailed simulations, including the direct calculations of the tunneling characteristics and optical gain, are necessary to optimize the structure design.

## Conclusions

Finally, the terahertz transitions between Landau levels of different subbands in resonant tunneling quantum well structures in a tilted magnetic field were considered. An effective way was proposed to lift the selection rule forbidding the intersubband inter-Landau level transitions by placing the structures into the tilted magnetic field. An importance of asymmetrical structure potential was revealed, and the possibility to achieve considerable values of inter-Landau level transition matrix element was demonstrated for an asymmetric double-well structure.

## Competing interests

The authors declare that they have no competing interests.

## Authors' contributions

MPT participated in the general task formulation and carried out the theoretical background and intersubband matrix element calculations. YAM of conceived the general task formulation, participated in the study design and coordination, and drafted the manuscript. PFK carried out the simulation program development and energy band structure calculations and participated in the sequence alignment. All authors read and approved the final manuscript.
